# Diagnostic Pathways as Social and Participatory Practices: The Case of Herpes Simplex Encephalitis

**DOI:** 10.1371/journal.pone.0151145

**Published:** 2016-03-09

**Authors:** Jessie Cooper, Ciara Kierans, Sylviane Defres, Ava Easton, Rachel Kneen, Tom Solomon

**Affiliations:** 1 Department of Public Health and Policy, University of Liverpool, Liverpool, United Kingdom; 2 Institute of Infection and Global Health, University of Liverpool, Liverpool, United Kingdom; 3 Tropical and Infectious Diseases Unit, The Royal Liverpool and Broadgreen University Hospitals Trust, Liverpool, United Kingdom; 4 The Encephalitis Society, Malton, North Yorkshire, United Kingdom; 5 Department of Neurology, Alder Hey Children’s NHS Foundation Trust, Liverpool, United Kingdom; 6 Department of Neurology, The Walton Centre NHS Foundation Trust, Liverpool, United Kingdom; 7 NIHR Health Protection Research Unit in Emerging and Zoonotic Infections, University of Liverpool, Liverpool, United Kingdom; University Hospital San Giovanni Battista di Torino, ITALY

## Abstract

Herpes simplex virus (HSV) encephalitis is a potentially devastating disease, with significant rates of mortality and co-morbidities. Although the prognosis for people with HSV encephalitis can be improved by prompt treatment with aciclovir, there are often delays involved in the diagnosis and treatment of the disease. In response, National Clinical Guidelines have been produced for the UK which make recommendations for improving the management of suspected viral encephalitis. However, little is currently known about the everyday experiences and processes involved in the diagnosis and care of HSV encephalitis. The reported study aimed to provide an account of the diagnosis and treatment of HSV encephalitis from the perspective of people who had been affected by the condition. Thirty narrative interviews were conducted with people who had been diagnosed with HSV encephalitis and their significant others. The narrative accounts reveal problems with gaining access to a diagnosis of encephalitis and shortfalls in care for the condition once in hospital. In response, individuals and their families work hard to obtain medical recognition for the problem and shape the processes of acute care. As a consequence, we argue that the diagnosis and management of HSV encephalitis needs to be considered as a participatory process, which is co-produced by health professionals, patients, and their families. The paper concludes by making recommendations for developing the current management guidelines by formalising the critical role of patients and their significant others in the identification, and treatment of, HSV encephalitis.

## Introduction

Encephalitis is a disease which causes inflammation of the brain tissue; it can be viral, bacterial or immune-mediated in origin [1]. In the UK, herpes simplex virus (HSV) is the most commonly identified infectious cause of encephalitis, with an annual incidence of between 1 in 250,000 to 500,000. The condition can have a devastating impact upon the lives of those affected, many of whom are left with a range of neurocognitive, social, and physical problems as a result of damage to the brain [2–5]. Beyond its immediate consequences for the individual, HSV encephalitis has a large impact on healthcare resources, due to costs associated with hospitalisation and rehabilitation; it also has important longer term consequences for patients, their families, and society, since fewer than 20% of adult sufferers will return to work [3, 5, 6, 7]. The longer term outcome for children is less well studied, but is likely to have similar consequences and even more impact on society due to the age of developing the illness.

Since the 1980s, mortality rates for patients with HSV encephalitis have been dramatically improved with the use of the anti-viral drug aciclovir [8]. As a consequence, however, the number of survivors with neuropsychological sequelae has actually increased. Research has shown that the prognosis for people with HSV encephalitis can be improved with early commencement of aciclovir therapy, as evidenced by poorer outcomes in patients given the drug more than 48 hours after admission to hospital [9, 10]. Achieving better outcomes for people with HSV encephalitis therefore requires prompt recognition, diagnosis, and management of the disease by healthcare professionals [6, 11]. Yet, owing to the initial non-specific presentation of the disease and the manifold nature of the diagnostic process, timely management is far from straightforward, as we outline below.

Firstly, the early symptoms of encephalitis, such as fever, headache, and mild confusion may mimic other, more common conditions, such as flu, and urinary tract infections; later neurological features such as speech disturbance and seizures are also often attributed to more common brain diseases, like stroke [12, 13]. This can lead to delays in the initiation of aciclovir whilst clinicians investigate better established causes of a patient’s symptoms. Secondly, reaching a diagnosis of encephalitis involves a diversity of specialist investigations. These include: performing a lumbar puncture (LP) to look for evidence of central nervous system inflammation and its cause, including to detect HSV DNA in the cerebral spinal fluid (CSF), and using neuroimaging, such as computed tomography (CT) and/or magnetic resonance imaging (MRI), to check for the presence of inflammation of the brain [6, 14]. Furthermore, these diagnostic processes are associated with their own problems. For example, neuroimaging and CSF findings can sometimes be normal in the initial days of the illness [5, 9, 15, 16, 17]. Taken together, these can lead to delays in establishing a diagnosis and initiating treatment for HSV encephalitis.

In response to the problems inherent in diagnosing and managing HSV encephalitis, UK National Clinical Guidelines for the management of suspected viral encephalitis in adults and children have been produced [6, 14]. The guidelines recommend empirical treatment with aciclovir if there is going to be a delay in performing the investigations, or where there remains a strong clinical suspicion of HSV encephalitis despite initial imaging or CSF findings being normal. In order to steer clinicians through the process, the guidelines are summarised in an algorithm, which outlines the recommended steps for identifying and treating viral encephalitis [6, 14].

However, like a map, care guidelines are abstracted from the routine practices and everyday experiences which underpin diagnostic work and disease management [18, 19, 20, 21, 22]. The guidelines for managing viral encephalitis do not, therefore, allow insight into medical work as it is practically accomplished within its everyday settings. Nor do they help us to understand how prospective patients and their significant others make sense of what is happening to them, or what they *do* in response to the problems being experienced. Studies of such activity—driven by social science research which takes the work of science and medicine as its focus—have demonstrated the importance of paying attention to routine practices, in order to understand how things like scientific facts or a medical diagnosis are produced [23, 24, 25, 26, 27]. Here, the term ‘practice’ refers simply to what people do: their “arrays of activity”, and the knowledge, interactions, settings, and objects which are bound up with them [28]. If we are to fully grasp the problems involved in the diagnosis and management of HSV encephalitis, it is therefore critical that we examine the practices and experiences which underpin these clinical processes.

Drawing on narrative interviews with people who were diagnosed with HSV encephalitis and their significant others, this paper will provide an account of the diagnosis and treatment of HSV encephalitis, in order to address two inter-related aims. These are: 1) to identify the specific concerns and practices of people with HSV encephalitis and their families, in response to their experiences of diagnosis and care; 2) to use these insights to make recommendations for creating a more responsive set of management guidelines and healthcare interventions, which could aid in improving the diagnosis and care for people with HSV encephalitis.

## Methodology

### Background to the study

The narrative interview data for this paper was collected between December 2012 and July 2014 as part of the ‘Understanding and Improving the Outcome of Encephalitis’ (ENCEPH-UK) applied research programme grant funded by the National Institute for Health Research (NIHR). The programme is made up of a series of inter-related cohort studies and a range of sub-studies, further details of which can be found at www.encephuk.org. The narrative data presented in this paper was collected for one of the programme’s sub-studies, called the *End User Experience study*. This aimed to develop a detailed understanding about HSV encephalitis and its management from the perspective of people who had been treated for the condition, as well as their significant others.

Narrative research is a form of qualitative inquiry which focuses upon the remembered account or story in order to explore how people recall, account for, and make sense of past events and actions which occur [29]. A narrative methodology largely, but not exclusively, utilises in-depth interviews which encourage people to develop an account of their experiences [30]. Additionally, narrative interviewing allows individual accounts to be situated within a wider social milieu, in this case, medical institutions or healthcare settings [31]. This enables the research to provide insight into the social structures and cultural contexts which underpin experiences, without losing the specificity of the individual narrative.

### Data collection

Participants were recruited to the *End User Experience study* from the main ENCEPH-UK cohort studies. These consisted of two groups of participants who were recruited from 60 hospitals across the UK: 1) a retrospective cohort who had encephalitis at any time between 2005 and 2012, and 2) a prospective cohort of patients who were recruited at the point of having suspected encephalitis in the hospital sites. The prospective paediatric patients in ENCEPH-UK were recruited via the Childhood Meningitis and Encephalitis study (UK-ChiMES); a collaborative study with the University of Oxford (www.encephuk.org/studies/ukchimes). Cases of neonatal HSV encephalitis were excluded. Due to the rarity of HSV encephalitis, additional retrospective participants were recruited by advertising through *The Encephalitis Society* in the UK.

Participants enrolled into the main cohort studies were purposively recruited to the *End User Experience* study on the basis of them having received a diagnosis of HSV encephalitis. Since it was important to understand how the experience of encephalitis had changed over time, participants were sampled across those who had recently been discharged from hospital (between 3 and 6 months, from the prospective cohort), and those who had been treated for encephalitis further in the past (between 1 and 8 years, from the retrospective cohort). Additionally, cases were varied between people treated in general hospitals and those who were managed in tertiary centres (specialist infectious diseases, neurology units, or paediatric centres).

Participants were initially contacted by phone or email by JC and were sent an information sheet about the study if they were interested in taking part. In total, 30 narrative interviews were conducted by JC with 45 participants in their own homes (17 interviews with 26 participants from the retrospective cohort, and 13 interviews with 19 participants from the prospective cohort); one participant from the prospective cohort subsequently withdrew from the study, making 29 transcripts available for the final analysis. Since people with encephalitis often had little memory of the time during their acute illness, many chose to be interviewed with a relative or friend who was present during their hospitalisation. Many of the accounts were therefore co-produced by encephalitis patients and their relatives. In the case of children under the age of 16, ethical constraints meant that their parents were recruited for interview (N = 5). Additionally, in two adult cases, interviews were done solely with their relatives. This was because in one case the patient had died, and in the other the patient had acquired severe neurocognitive problems, meaning she was unable to participate (Table 1).

**Table 1:** Participant characteristics and interview details of patients with HSV encephalitis.

Person with HSV encephalitis	Age at interview	Gender M/F	Type of hospital treated in [General hospital (GH) Tertiary hospital (TH)]	Interview details
***Retrospective Cohort***				
1	45	M	Admitted to GH, transferred to TH (neurology)	Interviewed with partner
2	47	F	Admitted to psychiatric hospital, transferred to GH	Interviewed with mother
3	43	M	TH (infectious diseases)	Interviewed with partner
4	58	M	Admitted to GH, transferred to TH (neurology)	Interviewed with wife
5	15	M	TH (paediatric neurology)	Interview conducted with the parents
6	62	F	GH	Interviewed alone
7	68	F	GH	Interviewed alone
8	55	F	Admitted to GH, transferred to TH (neurology)	Interviewed with friend
9	36	M	GH	Interviewed with wife
10	5	M	GH (paediatric)	Interview conducted with the child’s mother
11	56	F	Admitted to GH, transferred to TH (neurology)	Interview conducted with husband
12	20	F	TH (paediatric)	Interviewed alone
13	34	F	TH (neurology)	Interviewed with partner
14	55	F	TH (Infectious diseases)	Interviewed alone
15	6	M	GH (paediatric)	Interview conducted with the child’s father
16	33	M	Admitted to GH, transferred to TH (neurology)	Interviewed with mother
17	61	F	Admitted to GH, transferred to TH (neurology)	Interviewed alone
***Prospective Cohort***				
1	69	M	TH (neurology)	Interviewed alone
2	58	M	GH	Interviewed with wife
3	27	M	Admitted to GH, transferred to TH (neurology)	Interviewed alone
4	61	F	TH (infectious diseases)	Interviewed with husband
5	67	M	GH	Interviewed with wife and daughter
6	77	F	TH (infectious diseases)	Interview conducted with husband and son (patient died)
7	35	M	GH	Interviewed alone
8	58	F	GH	Interviewed alone
9	75	M	TH (infectious diseases)	Interviewed with wife
10	63	F	GH	Interviewed with sister
11	6 months	F	GH, temporarily transferred to TH (paediatric surgery)	Interview conducted with the child’s mother
12	2	M	TH (paediatric)	Interview conducted with the child’s mother

The study was approved by the National Research Ethics Service (NRES), East Midlands Committee (11/EM/0442). Written consent was taken from participants prior to each interview, and all interviews were digitally recorded and transcribed in full. Participants were assigned pseudonyms, and other identifying aspects of the accounts were anonymised during transcription. The interviews focused upon participants’ experiences of: diagnosis, which encompasses the experience of initial symptoms, diagnostic investigations, and admission into hospital; the care received in hospital; everyday life post-hospitalisation; and care received after discharge from hospital. Interviews followed an adapted version of Wengraf’s format for narrative interviewing and lasted between 20 minutes and three and a half hours [30]. Consideration was also given to the level of fatigue experienced by participants, for example, since people are more commonly fatigued in the first few months post-discharge, interviews tended to be shorter for participants who had recently left hospital.

### Analysis

Narrative inquiry is interested in privileging the way in which people make sense of the world around them, how they reflect on what they do within this world, and the context and production of meaning within narrative accounts. The narrative interviews for this study generated rich insight into the experience of diagnosis and treatment for encephalitis, and the processes involved in accessing and shaping amorphous care systems around the condition.

While the narratives demonstrated a diversity of experiences around these processes, the analysis was principally concerned with ‘structural commonalities’ across the accounts [32, 33]. This refers to the way in which the accounts emphasised, and were similarly shaped by, particular institutional constraints or modes of organisation: for example, how the diagnosis of HSV encephalitis was experienced as a particular issue in relation to the perceived lack of recognition for the problem from health professionals, and how people made decisions and took actions in the context of these limitations. To enable insight into these processes, we adopted a dual analytic approach [29]. This incorporated narrative analysis with a more general thematic analysis. The narrative analysis used an adapted version of Labov’s structural analytic approach [34, 35]. This focused upon examining the organisation of a narrative, in relation to how events were described and interpreted by the teller. The categories generated from the narrative analysis were then used as the basis for the thematic analysis. This was concerned with interpreting patterns across accounts, in terms of the common challenges which people were faced with when being diagnosed and treated for HSV encephalitis, and the kinds of strategies people employed in response, such as what they did when encountering particular symptoms. In so doing, we were able to characterise the conditions which give rise to the particular experiences across the narrative accounts as a whole.

In order to illustrate our findings, we present three encephalitis cases. While the cases all have their own idiosyncrasies, these cases were chosen due to their typicality in experiences across the dataset. Before turning to the results, it is important to emphasise that the narrative data we present is not seen as a ‘factual’ account of the events as they occurred, but instead, is a remembered account, where peoples’ interpretations and understandings are privileged [29, 36].

## Results

The three examples, below, relate to two retrospective encephalitis cases and one prospective case. The first retrospective case is of Stephanie, a woman in her 60s, who was diagnosed with HSV encephalitis in 2005, seven years prior to being interviewed. Stephanie lived by herself and had worked as a psychologist before taking early retirement after suffering from encephalitis. She told her story using notes she had produced from her own and her family members’ recollections of her time in hospital. The second retrospective case is of Greg, a man in his mid-30s with two young children. Greg was diagnosed with probable HSV encephalitis in 2012, 1 year prior to being interviewed. He was made redundant from his managerial job not long after his diagnosis, and, due to struggles with fatigue and memory problems, subsequently took a role with fewer responsibilities. Greg was interviewed with his wife, Nicola. The prospective case relates to Ben, a retired metal worker in his 70s, who had been discharged from hospital after being treated for HSV encephalitis four months prior to being interviewed. Ben told his story alongside his wife, Janet.

All three cases demonstrate concerns which are characteristic of the wider participant experiences around encephalitis diagnosis and treatment, and the various ways in which patients and their families respond to these experiences. In particular, these accounts reveal how people come to identify a serious medical problem with the realisation that they, or their relative, are feeling or acting out of character. The narratives highlight the subsequent difficulty involved in gaining medical recognition for the problem, along with the practical work many families must do to ensure that their relative receives treatment for the symptoms being experienced. The cases of Stephanie and Greg also illustrate that the problems do not end with the receipt of a diagnosis, but extend into experiences of hospital care and the care environment. As a result, families continue to work at shaping the processes of care around their relative’s needs. Taken together, these accounts illustrate the ways in which people with encephalitis and their significant others play a critical role in creating a diagnosis and managing the condition of HSV encephalitis. Each of the cases are detailed below.

### Stephanie

Stephanie first realised that something was wrong while she was out driving one day and started to feel “a bit distanced from reality and cool and separate”. Over the following few days she experienced headaches, strange “feelings of unreality” and became aware that her urine was a “peculiar dark colour”. Five days later, after realising these symptoms were persisting, Stephanie made an appointment with her GP. At the doctors, Stephanie felt that the examining GP had an “extreme disinterest” in her case: she took a urine sample and told Stephanie to wait a week for the results. Stephanie remembered “sitting there feeling unreal, aware that I really was in need and was ill, and being told quite rudely [by the GP] to get up and go”. Nine days after Stephanie first experienced symptoms, her daughter, Sarah, worried about the lack of contact from her mother, tried to telephone her. When Stephanie answered the phone in an incoherent manner, Sarah was sufficiently concerned to call an ambulance.

Once in hospital, Stephanie was told by her daughter that she spoke in a “peculiarly over simplistic fashion” to the examining doctor, who saw her ability to speak as a good sign. However, her daughter made it clear that this manner of speech was totally out of character for her mother: who worked as a psychologist and was a very articulate woman. That evening, doctors told Stephanie’s children that she was probably suffering from either meningitis, septicaemia, or, the least likely, encephalitis, and she was given antiviral medication as a precautionary treatment for encephalitis. Stephanie had a lumbar puncture the same day, and the result confirmed a diagnosis of HSV encephalitis a week later.

Using her notes, Stephanie detailed how her family were a constant presence on the ward during her time in hospital. Sarah drew up a family rota, and made sure that someone was with Stephanie every day. She also supplemented the formal care of staff by making her own notes about Stephanie’s medical charts and staying overnight with her mother when the ward was understaffed. Stephanie’s brother, a GP, sought out information about encephalitis by consulting his colleagues and staff on the ward. His acquired knowledge about the condition meant he was able to dispute the decision of doctors regarding the time-frame that Stephanie should be kept on aciclovir. Despite these issues, Stephanie was keen to highlight the care given to her by staff on the ward, who she described as “wonderful”. As an example, Stephanie recounted having “horrendous” dreams one evening, and the comfort given to her by one of the nurses, who sat with her, held her hand, and told her to concentrate on the pictures of her family pinned to the wall. Reflecting on this incident, Stephanie explained how: “that was powerful treatment; it wasn’t just comforting it was actually really powerful”.

Two weeks after being admitted to hospital, Stephanie was discharged home.

### Greg and Nicola

Nicola explained that the first indication that something was wrong with Greg was when he arrived home from work one evening and told her he was tired and had a headache; she also noticed he seemed wobbly on his feet. The next day, after “stumbling” about the house and slurring his speech, Greg told Nicola that he was feeling unwell. Since Greg was not someone who usually complained when he was ill, Nicola was suitably worried and made him an appointment with the GP. In the GP consultation, Nicola explained that Greg kept on “disappearing into a world of his own” and she expressed her fear to the doctor that he might be suffering from meningitis. Concerned, the GP sent Greg to the Urgent Care Unit at his local hospital. Here, Greg was assessed for a stroke, which Nicola perceived as a mistake—since he was not exhibiting the signs of stroke, which she understood to be a drooping face and weakness on one side. When Greg’s CT scan came back normal, he was sent home to wait for an “urgent” MRI scan. The next day, Nicola became increasingly alarmed at Greg’s behaviour, recalling how she: “couldn't rouse him, short of probably punching him in the face he probably wouldn't have snapped out of it”. Unhappy with the situation, she took him back to the Urgent Care unit and insisted that they do something, telling them: “he’s not right, he’s got worse. I’m not taking him home until you find out what’s going on with him”.

Greg was started on aciclovir that evening and placed in an overflow ward; it was explained to Nicola that he had suspected viral encephalitis. In reflecting on that evening, Greg described feeling distressed at the “horrendous” conditions on the ward: the bright lights, disgruntled staff, and constant noise from elderly patients compounded his splitting headache. To make matters worse, Greg and Nicola experienced an inconsistent approach to his care. Greg explained that, a few days after he was admitted, the doctors made an “incredibly stupid decision” and stopped his medication. Two days later, after he complained of feeling unwell again, Greg was told he would need to go back on aciclovir. However, by that evening he was still waiting for the medication, and Nicola complained to the ward staff about the lack of action. She reflected on this delay in terms of the possible consequences it could have caused: “You can't say it takes 10 hours to get an antiviral medication for a patient that is potentially going to be left with a more serious brain injury if you don't give it to them soon”. Greg and Nicola were also frustrated at the poor communication from staff about Greg’s diagnosis and care plan. As a result, Nicola took it upon herself to research viral encephalitis on the internet, and requested that doctors write down answers to her questions, so that Greg could process the information in his own time. She described how her request was ignored, and doctors continued to provide information orally to Greg. Less than a week into his stay in hospital the conditions on the ward meant that Greg had “had enough” and he tried to discharge himself before his treatment had finished. He was subsequently allowed home, with aciclovir continued via a home treatment team.

### Ben and Janet

Ben first experienced “strange” symptoms when he was out walking his dog one day and began to fall over. He knew something was amiss since he was not generally prone to clumsiness. Ben described feeling “really odd” after his walk, and told Janet that he thought there was something wrong with his brain. Later on that day, Janet found Ben slumped in a chair; assuming that he had had a stroke she took him to the GP and told the doctor she thought Ben should go to hospital. When the GP examined Ben he was sufficiently concerned to call for an ambulance. However, when the paramedics arrived, they disputed the doctor’s suspicion of a stroke. They questioned Janet about whether Ben had suffered headaches or been sick, and told her they thought he had a norovirus infection. Unsure of this diagnosis, Janet questioned the paramedics’ judgement, asking them whether norovirus would “cause confusion and sleeping a lot, and falling down. And he [the paramedic] said ‘oh yes it can cause all that’”.

Once at the hospital, Ben was put into an isolation unit where he was told he would have to wait to be admitted because there was a lack of available beds. In the early hours of the morning, Ben called Janet to tell her he had been discharged after the doctors had said there was nothing wrong with him. Janet was incredulous at the decision, and Ben recalls how he felt as if he was “in cloud cuckoo land”, and found it very difficult trying to call a taxi to get home. In the days which followed, Ben was, according to Janet, “out of it” and spent much of the time asleep. Unhappy with his lack of improvement, Janet called the GP who said he would see Ben the next day, and assured Janet that he would refer him back into hospital. That evening, Ben tried to get up to go to the toilet; his last memory of that day is of falling over when he tried to get out of bed. Janet later discovered Ben collapsed on the floor and immediately called an ambulance.

In the hospital, doctors informed Janet that they suspected an infection on Ben’s brain. This prompted Janet’s memory, who suggested a possible link to the cold sore virus to the doctor, explaining how “years and years ago I had read in a book or a paper that a cold sore had killed a woman, and I used to get loads of cold sores so I always worried about [it], so when he [the doctor] had said that [about the brain infection] I just said to him, ‘it couldn't be to do with a cold sore could it?’ Anyway the doctor come back and he said thanks for that [suggestion] because we could have been looking [for a diagnosis] for a long time.”

## Discussion and Conclusion

The cases presented above describe typical processes by which the diagnosis and care of HSV encephalitis is experienced, and, significantly, gets *co-produced* by the work of patients and their families. Specifically, these processes include: 1) how a serious medical problem comes to be identified by individuals and their families; 2) the practical work families must do to get medical recognition for the problem and obtain a diagnosis and treatment; 3) the associated experiences of care for encephalitis within hospitals; and 4) and the subsequent efforts of families to organise clinical care around the needs of their relative. These processes can, in turn, be mapped on to two interlinked stages in the experience of the diagnostic and treatment trajectory for HSV encephalitis, namely: 1) access to diagnosis, and 2) care within acute settings. The processes are explained in detail below, under their respective stages within the diagnosis and treatment trajectory. We then discuss the significance of these findings for clinical practice and for the development of the management guidelines for viral encephalitis.

### 1. Access to diagnosis

#### Situated concern: the recognition of a serious medical problem

Most participants [23/29 (80%)] described the amorphous character of the symptoms experienced or witnessed at the outset of the illness. These ranged from: fever, feeling generally unwell, headaches, vomiting/nausea, tiredness, and disorientation, which were often attributed to more well-known causes, like the flu. In over half of cases [16/29 (55%)], the recognition that something was seriously wrong was felt by the sufferer or noticed by their significant others when something was seen as being amiss in their usual character or behaviour. In other words, peoples’ concerns were situated around an intimate knowledge of what was ‘normal’ for themselves or their relative. For patients, this was often described as feeling generally “strange”, “odd”, or “distanced” from their usual self; in Ben’s case, he knew that something was wrong when he began to trip up, since he was not prone to falling over. Similarly, the problem was noticed by relatives when their family member started acting out of character and were seen as being “out of it”, for example, by not being able to communicate properly; in Greg’s case, Nicola described how he was uncommunicative and “in a world of his own”. There was therefore an understanding that the person was dissociated from their usual self and unable to participate in their usual routines.

The reasoning by individuals and their significant others that something was seriously wrong was related to their subsequent attempts to get help from the medical profession. This involved using primary care services, such as visiting the local GP, phoning NHS helplines, and attending walk-in centres; in some cases people also visited the Emergency Department (ED) department of their local hospital. During initial consultations with health professionals, people reflected on how symptoms were, in the first instance, usually attributed to non-urgent conditions, like the flu, and they were often told there was nothing to worry about. Upon reflection, patients and their significant others interpreted the misrecognition of their symptoms as not having their concerns taken seriously by health care professionals. This is illustrated by Stephanie’s account, when her need for help was met with, what she perceived to be, disinterest from her GP. The initial lack of clinical recognition for the problem at-hand led to families needing to work at getting a diagnosis, efforts we describe below.

#### Persistent action and persuasion work: the making of an encephalitis diagnosis

In those cases [9/29 (31%)] where there was a perceived lack of adequate action from health professionals, and as peoples’ symptoms worsened, family members took matters into their own hands. People persisted in bringing their relative’s case to the attention of health professionals by: making multiple phone calls and/or visits to the GP, communicating their concerns to a range of NHS staff, including GPs, nurses, doctor’s receptionists, paramedics, and ED clinicians, and demanding that health professionals take their worries seriously by refusing to leave until diagnostic investigations were done, as we saw with Nicola, who refused leave Urgent Care until action was taken about Greg’s situation. Relatives also tried to persuade health professionals of the seriousness of the situation by, firstly, drawing on their intimate knowledge of the person to reinforce the fact that their behaviour was out of character. For example, Stephanie’s daughter insisted to the doctor that her mother did not usually speak in such a simple manner, after it appeared that the clinician was taking her ability to speak as a good sign. Secondly, family members utilised external sources of information to inform their conversations with health professionals. This included talking to family and friends about the situation, consulting the internet, and drawing on their existing knowledge of other serious conditions, such as stroke and meningitis. In two cases this also involved directly suggesting the possibility of HSV to health professionals, as we saw with Janet describing a magazine article about a cold sore leading to a brain infection. By persisting in attempts to persuade healthcare professionals of the seriousness of the situation, relatives worked hard to gain access to a diagnosis of encephalitis for their family member.

Achieving a diagnosis of HSV encephalitis was therefore often seen as reliant on the way in which participants’ interpreted symptoms as warranting attention, and their subsequent efforts to gain medical recognition for them. In contrast, when an early encephalitis diagnosis did occur, this was often understood by participants as happening on an ad hoc basis, such as being seen by a doctor with previous experience of encephalitis, or a GP concerned about related neurological conditions, like meningitis or stroke. In some cases, early identification of the condition was understood to be due to the obvious manifestation of a medical emergency: when the person had a seizure or collapsed. Here, the seriousness of the situation was immediate and mobilised an urgent medical response. However, for some of our study’s participants [9/29 (31%)], this emergency response occurred after the initial work of patient/relatives to try and get medical help. This was illustrated in Ben’s case, who was sent home from hospital after Janet relayed her concerns to both the GP and paramedics. It was only once Ben collapsed that his situation was recognised as a brain infection by doctors.

The work which is done by individuals and their families to identify and gain medical recognition is therefore clearly critical to the process of achieving a timely diagnosis of HSV encephalitis, a point which we discuss later in more detail. The involvement of families did not stop at accomplishing a diagnosis, however. As we explain below, the participation of significant others continued into the provision of acute care in hospital.

### 2. Care within acute settings

#### Experiences of care and treatment for encephalitis

Many participants [19/29, (66%)] claimed that aspects of their care once in hospital and in receipt of a diagnosis were unsuitable for their or their relative’s particular needs. Three principle concerns raised by participants involved: **1) inappropriate care environments:** many people were treated in general wards, which were understood as not accommodating of their need for quiet, dark conditions; non-specialist staff were also perceived to lack knowledge about encephalitis and how to care for people with the condition [37]. This was shown in Greg’s case, who felt that the conditions on the overflow ward compounded his suffering; others described how bright lights on the ward exacerbated their/their relative’s symptoms; **2) Communicative gaps**: Participants also experienced poor communication from hospital staff about the diagnostic investigations being done to them/their relative, what encephalitis was, and its treatment and prognosis. These communicative gaps were viewed as particularly frustrating since, for most participants, encephalitis was an unfamiliar condition at the time of diagnosis. This meant they did not have existing knowledge about the disease to help them grasp what was happening to them/their relative. Furthermore, when information was provided by healthcare staff, this was understood to sometimes fall short of the needs or capacities of patients. For example, in Greg’s case, Nicola’s request for doctors to write down information for Greg was ignored, meaning he was unable to remember the oral information that was given; **3) Care deficiencies:** participants experienced inconsistencies and poor management in the care around HSV encephalitis. These were articulated in terms of perceived mishaps or oversights in the care given to patients. For example, treatment with aciclovir was halted without explanation, test results were delayed or mixed up, and medical complications went unnoticed or untreated.

These experiences of care inadequacies can be characterised as institutional limitations, in that they are tied into: existing ways of organising patient care, a lack of understanding of the nature of HSV encephalitis, and the problems experienced by patients suffering from the condition. In particular, this includes the lack of a suitable ward environment for people with “splitting headaches” and light sensitivity, and the problems associated with communication about a condition which was a) unfamiliar to most, and b) directed towards patients with neurological difficulties.

In contrast, although nearly half [12/29 (41%)] of interviews narrated some aspects of good care, this was articulated in more general terms by participants, in that it could be related to the care of *any* acute condition. Specifically, positive experiences of care were characterised in terms of the particular attentiveness which was given to patients and their families. This was expressed in two main ways: **1) personalised care:** participants recounted certain incidents of care, or staff members who provided comfort and emotional support about the situation to them, as individuals. This is illustrated by Stephanie’s account, who recalled an episode in which she was helped out of her distressed state by a particularly vigilant nurse; **2) communication that counts:** in contrast to the poor communication experienced by participants, a few relatives detailed the effective and supportive communication given to them by hospital staff. This included a) being given transparent accounts of what was happening to their family member and the treatment options, and b) having information delivered in a way that was understandable and reassuring. For example, the mother of a toddler recalled the effective way in which doctors explained what was happening to her son, who had a stroke as a result of encephalitis: “they told me that it’s like a traffic jam inside the body and it’s stopped the blood flowing (…) I just thought what a good way to explain it to people that are really upset”.

In summary, despite the fact that people highlighted aspects of good care in hospital, this was usually isolated to certain incidents of care, or care given by individual members of staff. In general, participants focused upon intrinsic problems with the overall care received and, in turn, reflected on the practices they adopted in response to these limitations, as we explain below.

#### Re-articulating care systems

In those cases [19/29 (66%)] that experienced inadequacies in the systems of care for HSV encephalitis, family members would often [13/19 (68%)] take it upon themselves to ‘re-articulate’–re-organise and adjust—the care that their relative was receiving into a form that fit their needs [36]. This was done in two, interlinked ways. Firstly, since most patients were unconscious or incapable of articulating their own needs while in hospital, family members became guardians of their relative’s wellbeing by developing their own systems of vigilance. This was done by effectively becoming the eyes and ears for the patient and overseeing how they were being treated. These informal surveillance systems involved: 1) gathering information about their relative’s care and HSV encephalitis in general, by reading and taking notes of the patient’s medical charts, searching the internet, reading books, and appealing to hospital staff for further information; 2) ensuring there was a regular presence of family and friends at the patient’s bedside by organising rotas and mobilising the support of social networks.

Secondly, these forms of vigilance enabled family members to draw attention to, what they experienced as, inadequacies in care and to work at adjusting this care to make it appropriate for their relative’s needs. This re-articulation of care was achieved by family members drawing on an array of tactics, which involved: making formal and informal complaints to staff about the perceived gaps or faults in care; using the information they had gathered to define the forms of medical intervention and resources they thought were needed; and becoming actively involved in clinical decision-making about their relative’s care. Tactics also included making adjustments to aid their relatives comfort, such as by bringing in objects from home, and filling in the gaps in care, as with Stephanie’s daughter who stayed overnight with her mother when she felt there were staff shortages. Taken together, these tactics ensured that formal systems of care were informally shaped around the specific requirements of encephalitis patients, in order to make sure that their needs were adequately met.

### Implications of the findings for encephalitis diagnosis and care

This paper has shown that people with HSV encephalitis and their significant others play a critical role in the diagnosis and treatment of HSV encephalitis. Specifically, they are crucial to: a) identifying that there is a serious medical problem, and b) providing a route by which a diagnosis can be made. While previous social science research has emphasised the importance of patients and their significant others in shaping patient pathways, or what has been termed ‘illness trajectories’ [38, 39, 40], this work has gone further to reveal the specific contribution that patients and families can play in helping to forge a medical diagnosis.

The route to achieving a timely diagnosis of HSV encephalitis, and, ultimately, improving patient outcomes should therefore be understood as a participatory process. In other words, the intimate knowledge of individuals and their families that something is amiss, and their subsequent actions to get help can be as crucial to diagnosis as the practices and decisions of health professionals [23]. Moreover, the role of families in facilitating a medical response to HSV encephalitis is not isolated to diagnosis: it continues into acute care and the work often required of families to adjust the care around the specific needs of their relatives. Taken together, these results have obvious implications for improving the practices involved in the diagnosis, treatment, and ongoing care for people with HSV encephalitis.

Recommendations for practice could be incorporated into the existing National Clinical Guidelines, and also be used to inform the production of a care pathway for HSV encephalitis; they involve recognising the vital work of individuals and their significant others in encephalitis management in two ways. Firstly, by taking seriously their accounts when individuals and families judge behaviour that is clearly out of character or has shifted from ‘normality’. While management guidelines emphasise altered behaviour and personality as one of the clinical features that should lead to suspicion of encephalitis, they do not necessarily acknowledge the critical role played by patients and families in recognising, and drawing attention to these changes. We therefore recommend the use of a key diagnostic question when individuals/families express such concerns: *what is normal for you/your relative*, *and how have things/has their behaviour recently differed from this*? This question could be asked not only by hospital clinicians but also by GPs and paramedic staff, who act as key gatekeepers to the provision of urgent assessment in hospital. Asking this question of patients and families may go some way to ensuring more timely recognition of the problem. Secondly, the action of families in shaping the course of acute care highlights the need to formalise the role of relatives and carers in the ongoing care for people with encephalitis. We therefore recommend that multidisciplinary treatment teams (e.g. clinicians, nurses, neurologists, physiotherapists, etc) should consider families as active team members in the process of caring for patients with HSV encephalitis, for example, by taking seriously their concerns and suggestions for patients’ needs around communication and comfort, and including them as participants in the decision-making processes of care. In so doing, this would also enable the creation of more personalised care plans, and the practical implementation of care more fully attentive to the individual needs of each patient, something which was highlighted as a key aspect of ‘good’ care within the accounts.

Recognising the roles of patients and their families in these ways, and developing the existing National Clinical Guidelines for viral encephalitis to include these aspects, may enable more a more effective approach towards the diagnosis, management, and ongoing care for people with HSV encephalitis, which would ultimately aid in improving patient outcomes.

There are a number of potential limitations of this study, and avenues for further research which need to be further explored. Firstly, since the research participants all had help from friends and family, this leaves the question of what happens to people who do not have significant others to advocate on their behalf. Future research could therefore examine how the experiences and processes of care might differ for this group. Secondly, our findings incorporated the experiences of a small number of parents whose babies and children suffered from HSV encephalitis. Whilst the recommendations for practice could obviously be applied to the management of HSV encephalitis in paediatric settings, the small sample size of parents and the variety of ages of the paediatric cases (from six months to 15 years) make it difficult to draw conclusions about the mechanisms involved in the diagnosis and care for this sample. There is a need, therefore, to conduct further research into these experiences from the perspective of parents; this would include sampling cases across a range of ages, to understand how the processes of diagnosis and treatment can be characterised for paediatrics. Details of one of the paediatric cases in this study can be found in a previous publication [41]. Thirdly, since the results in this study relate specifically to HSV encephalitis, we are not able to comment on the generalisability of the experiences to other patient populations. However, since the findings highlight institutional issues in the diagnosis and treatment of HSV encephalitis, it is plausible to suggest that they may help to make sense of, and open up questions about, the processes involved in the clinical management of other acute neurological conditions [42, 43]. Finally, a further limitation relates to how the narratives did not focus in detail on the diagnostic investigations for encephalitis, such as the experience of having a lumbar puncture. The fact that these processes were not often discussed indicates that they were not well remembered by participants, perhaps because they were overshadowed by struggles to gain recognition for symptoms. This limitation also extends to the fact that the study did not directly observe the interactions between healthcare professionals, patients, and families. Future ethnographic research is therefore needed, to extend the insights of this study by observing the processes of HSV encephalitis diagnosis and management as they occur.

Expanding the research insights in these ways would further our aim to create more responsive guidelines and healthcare interventions to improve the care of, and outcomes for people with the potentially devastating condition of HSV encephalitis.
